# Inflammatory, transcriptomic, and cell fate responses underlying the mammalian transmission of avian influenza viruses

**DOI:** 10.1128/jvi.00647-25

**Published:** 2025-08-08

**Authors:** Mark Zanin, Timothy Flerlage, Sook-San Wong, Peter Vogel, Kristine Piza, Patrick Schreiner, Zhongshan Cheng, David F. Boyd, Rabeh El-Shesheny, Jeremy C. Jones, Ti-Cheng Chang, Paul Thomas, Robert Webster, Richard Webby

**Affiliations:** 1 School of Public Health, LKS Faculty of Medicine, The University of Hong Kong 25809, Hong Kong SAR, China; 2 Center for Immunology & Infection, Hong Kong SAR, China; 3 Department of Infectious Diseases, St. Jude Children’s Research Hospital 5417, Memphis, Tennessee, USA; 4 HKU-Pasteur Research Pole, LKS Faculty of Medicine, The University of Hong Kong 667036, Hong Kong SAR, China; 5 Veterinary Pathology Core, St. Jude Children’s Research Hospital 5417, Memphis, Tennessee, USA; 6 Department of Host-Pathogen Interactions, St. Jude Children's Research Hospital 5417, Memphis, Tennessee, USA; 7 Center for Applied Bioinformatics, St. Jude Children's Research Hospital 5417, Memphis, Tennessee, USA; 8 Department of Molecular, Cell & Developmental Biology, University of California 8787, Santa Cruz, California, USA; 9 Center of Scientific Excellence for Influenza Viruses, National Research Centre, Cairo, Egypt; 10 Department of Immunology, St. Jude Children's Research Hospital 5417, Memphis, Tennessee, USA; Emory University School of Medicine 12239, Atlanta, Georgia, USA

**Keywords:** avian influenza virus, transmission, ferret, nasal turbinate, zoonosis, single-cell transcriptomics

## Abstract

Airborne transmissibility of avian influenza viruses (AIVs) in humans is considered an essential component of their pandemic risk. Although several viral factors regulating airborne transmission (AT) have been delineated, it is not known what, if any, responses at the respiratory epithelia are determinants of AIV AT. Using responses in the ferret nasal epithelium to a panel of H1N1 AIVs, we describe host responses that segregate with AT phenotypes. AIV infection upregulated interferon alpha and gamma responses and IL-6 JAK-STAT signaling and downregulated oxidative phosphorylation. Single-cell transcriptomics revealed that cellular genotoxic stress and NF-kB, interferon, and cell fate pathways differentiated host responses to AIVs with different transmissibilities. These responses culminated in greater AIV antigen-containing exudate and debris in the respiratory spaces of the nasal epithelium of ferrets inoculated with AT AIVs. More abundant CMPK2, SP100, and CXCL10 transcription in infected epithelia was a hallmark of AT viruses. Overall, our study reveals host responses associated with AIV infection and transmission in the nasal epithelium, the determinant anatomical site of influenza virus transmission.

**IMPORTANCE:**

Airborne transmission (AT) is a critical component of the pandemic risks posed by avian influenza A viruses (AIVs). However, the host responses ultimately dictating transmissibility elicited by AIVs in the upper respiratory tract of mammals, the determinant site of influenza virus AT, are largely unknown. We identified host responses in the nasal epithelium of the upper respiratory tract differentially expressed in response to infection by AIVs of different mammalian ATs. Our data indicate that a definable host response was associated with AT of AIVs. These data would serve as an important basis for future mechanistic studies of AIV zoonosis and potentially have implications for understanding the mechanisms of transmission of respiratory viruses between humans.

## INTRODUCTION

Avian influenza A viruses (AIVs) maintained in the environment in aquatic birds pose a risk of zoonotic transmission to mammals. Studies of AIVs isolated from migratory birds have revealed that, often, a majority of these viruses can infect and cause disease in the murine model of influenza virus mammalian pathogenicity (1–7). Furthermore, the capacity for mammalian transmission also appears to be present, which is a critical factor in the pandemic potential posed by AIVs. Studies using the ferret model of human IAV transmission revealed that direct contact transmission appears to be surprisingly common and that some viruses also demonstrate airborne transmission (AT) (3, 8, 9). However, it is not known what, if any, host responses at the upper respiratory tract epithelia, the critical anatomic site for AT, are determinants of AIV AT (10, 11).

To gain insights into host-virus interactions associated with AT in the upper respiratory tract during AIV infection, we studied a group of subtype H1N1 AIVs isolated during long-term surveillance at Delaware Bay, USA, a critical stopover site for shorebirds during long-distance northward migration to arctic breeding grounds (12). This group of viruses all showed direct contact transmission in a ferret model and a subset also showed AT in this model (8, 9). Pathology studies of the respiratory tracts of ferrets inoculated with these viruses revealed that the upper respiratory tract, particularly the olfactory neuroepithelium, was the only site in the respiratory tract where tissue pathology differed between animals inoculated with AIVs of different AT phenotypes (9). Therefore, these findings revealed not only the mammalian transmissibility potential of AIVs isolated from wild aquatic birds but also the different host responses in the upper respiratory tract that may be determinants of AIV AT. As such, here, we studied host responses in the ferret upper respiratory tract to AIVs of differing mammalian AT potentials to gain insights into those responses that underlie AIV AT, which is a critical determinant of the pandemic potential of these viruses.

## MATERIALS AND METHODS

### Cells and viruses

Virus stocks were prepared in 9- to 10-day-old specific pathogen-free embryonated chicken eggs and titrated in eggs to determine 50% egg infectious doses (EID_50_). The viruses used were A/ruddy turnstone/Delaware/300/2009 (H1N1) (DE300), A/ruddy turnstone/Delaware/AI09_256/2009 (H1N1) (DE256), A/shorebird/Delaware Bay/558/2006 (DE558), and A/ruddy turnstone/Delaware/300/2009 (H1N1) containing S213P in NS1 (DE213). DE300 and DE213 were generated by reverse genetics (rg) as described previously (9).

### Animal experiments

Three-month-old male ferrets that tested seronegative for influenza virus were used in these experiments (Triple F Farms, Sayre, PA). Four ferrets were inoculated with 10^6^ plaque-forming units (PFU) of virus in 0.5 mL PBS (0.25 mL per nostril) following light anesthesia with isoflurane. Two ferrets were inoculated with 0.5 mL PBS (0.25 mL per nostril) as controls. Symptoms of influenza, including weight loss, increase in body temperature, and clinical signs (sneezing, lethargy, and ruffled fur) were recorded daily. Nasal washes were not collected to avoid disruption to the nasal epithelium. For tissue harvesting, ferrets were euthanized using pentobarbitone according to institutional protocols. Inhalation anesthesia was not used. Nasal turbinate tissues were collected and placed in ice-cold RPMI-1640 media (Gibco). Cells were isolated from the collected tissues using a MACS Epidermis Dissociation Kit (Human) (Milltenyi) and a GentleMACS dissociator according to the manufacturer’s instructions. Dead cells were removed using the MACS Dead Cell Removal Kit (Milltenyi) according to the manufacturer’s instructions.

### RNA sequencing analysis

Sequencing read quality was assessed using fastqc (13). Reads were aligned to the ferret genome MusPutFur1.0.96 using STAR and BWA (14, 15). Read counts were quantified using HTSEQ (16). Normalization and differential expression analysis were performed using limma-voom (17). Log_2_ TMM-normalized counts per million values were used to assess gene set enrichment in Hallmark gene sets from MSigDB (18, 19).

### Read alignment, coverage analysis, and Shannon Entropy analysis for viral RNA-seqs

Paired reads for each bulk cell RNA-seq were merged and trimmed before being aligned to the reference sequence (viral strain-specific reference sequence plus ferret cDNA sequence) using BWA mem. Of the 30 samples, 19 passed quality control. Each aligned bam of these 19 samples was further adjusted for read alignment with GATK. To evaluate the coverage of the eight viral genes in each bam, only viral reads that mapped to viral genes were extracted and saved into bam format. This read-cleaning step was necessary to evaluate coverage with viral reference sequences but not ferret sequences. These newly generated viral bams were then subjected to coverage analysis. The total number of reads located in a 50 bp window was normalized with the total number of reads mapped to the viral genes, the results of which were illustrated in coverage plots. Viral complexity (Shannon Entropy analysis) for each cleaned RNA-seq Bam was determined using quasitools based on viral-specific reference sequences. For each sliding window (200 bp) of each gene, Shannon Entropy values were determined. Viral mutations (including biallelic sites and sites with >2 alleles) were called using quasi-tools using viral corresponding reference, and these mutations located in the Shannon Entropy hot spots were identified.

### Single-cell transcriptomics

Following treatment with the Dead Cell Removal Kit, cells isolated from ferret nasal turbinates were counted and adjusted to achieve a targeted cell recovery of 10,000. Cells were then loaded into gel-in-emulsion (GEM) beads using a 10× Chromium Controller (10× Genomics, 1000204) and the Single Cell 3' GEM, Library & Gel Bead Kit v3 kit (10× Genomics, 1000075). After GEM loading, RNA was reverse-transcribed, incorporating cell-specific oligonucleotide tags. GEMS were then disrupted, and cDNA was amplified and used to create a cDNA library using library construction kits (10× Genomics, 1000075). cDNA libraries were then sequenced on an Illumina HiSeq sequencer with 100 bp length in the 5’ configuration. Library quality was assessed using the 2100 Bioanalyzer with the high-sensitivity DNA kit (Agilent Technologies).

### Single-cell transcriptomics analysis

We created a custom reference genome that included the *Mustela putorius furo* (domestic ferret) genome (ASM1176430v1.1, RefSeq GCF_011764305.1) in addition to individual influenza gene segments. These included (along with their GenBank accession codes) the following: (i) DE558 PB2 (CY137649.1), PB1 and PB1-F2 (CY137648.1), PA and PA-X (CY137647.1), HA (CY137642.1), NP (CY137645.1), NA (CY137644.1), M2 and M1 (CY137643.1), and NEP and NS1 (CY137646.1); (ii) DE256 PB2 (CY145930.1), PB1 and PB1-F2 (CY145929.1), PA and PA-X (CY145928.1), HA (CY145923.1), NP (CY145926.1), NA (CY145925.1), M2 and M1 (CY145924.1), and NEP and NS1 (CY145927.1); (iii) DE300 PB2 (KF424127.1), PB1 and PB1-F2 (KF424128.1), PA and PA-X (KF424129.1), HA (KF424130.1), NP (KF424131.1), NA (KF424132.1), M2 and M1 (KF424133.1), and NEP and NS1 (KF424134.1). rgDE213 NS1 differed from DE300 NS1 at T637C, encoding the amino acid change S213P. Individual single-cell cDNA libraries from each infected ferret were aligned to this custom reference genome and then aggregated without normalization using CellRanger (v7.0.0, 10× Genomics) for downstream quality control analysis in Seurat (v4.1.1).

Using the aggregated data, a Seurat object was created that includes features (or genes) detected in at least three cells, with each cell having a minimum of 200 features. Quality control (QC) metrics were assessed, and associated plots were constructed to visually explore the quality of the data (20). Metrics used for assessments were the following: cell counts, number of unique molecular identifier (UMI) counts per cell, number of genes detected per cell, number of genes vs. number of UMIs detected, mitochondrial counts ratio, and number of genes per UMI. Poor-quality cells were filtered out. The mitochondrial ratio, which gives a ratio of cell reads (or counts) from mitochondrial genes, was used to check mitochondrial contamination from dead or dying cells. We defined poor-quality cells as having a mitochondrial ratio of more than 0.2. To better compare the samples, data were log_10_ transformed. The “complexity” of the data set could be determined by the number of genes detected per UMI—the greater the number of genes detected per UMI, the more complex the data. Generally, the score for overall complexity of gene expression is expected to be above 0.80. The combined effects of the different metrics were considered in setting thresholds to avoid filtering out biologically important cell populations. Thus, to keep high-quality cells without removing biologically relevant cell types, data were filtered by excluding cells with less than 250 genes, less than 500 UMIs, less than 0.8 log_10_ genes per UMI, and cells with mitochondrial ratios higher than 0.2.

After QC, we performed clustering to separate different cell types into unique cell clusters. Before clustering, SCTransform was done to normalize data and perform variance stabilization (21). Unwanted variations due to mitochondrial expression, cell cycle scores, and influenza gene segments were regressed out. Data were then analyzed with the top principal components (PCs) to ensure that most of the variation was captured. The elbow plot was used to visualize the standard deviation of each PC. We also used a more quantitative approach with the elbow plot to determine the top PCs. We then used 18 PCs for Uniform Manifold Approximation and Projection (UMAP) and cell clustering with Seurat’s graph-based clustering approach.

Further analyses, including cell population annotation, differential gene expression (DGE) testing, and pathway enrichment analysis took place in Seruat (v4.0.3) in the R statistical computing environment (v4.0.2). For subclustering, dims/resolution in Seurat functions RunUMAP, FindNeighbors, and FindClusters was set to 1:12/0.2 for IAV (Fig. 3D), 1:15/0.4 for innate (Fig. 3F), and 1:14/0.4 for the initial mono/mac/gran subcluster prior to removal of contaminating cells for additional analysis (Fig. S1I). Dimensions for reduction and visualization were chosen after a review of the Seurat function ElbowPlot for each subclustered cell population. Cellular annotation was performed manually by comparing the results of DGE testing for individual cell populations with previously published literature (22, 23). DGE testing was performed with the DefaultAssay = SCT using the Seurat function FindMarkers in default settings, which performs a Wilcoxon Rank Sum test between two groups of cells. Pathway enrichment analysis was performed with the DefaultAssay = SCT using the Seurat function DeEnrichRPlot, which identifies differentially expressed genes between two groups of cells using a Wilcoxon Rank Sum test, in default settings with enrichR = MsigDB_Hallmark_2020, max genes = 2,000, num.pathway = 10, and balanced = F). Influenza gene segment module scoring was performed using Seurat’s AddModuleScore with a list of the influenza gene segments included in the custom reference to which data were aligned (note we did not detect DE256-PB1 in the data set). Violin and dot plots were created with the DefaultAssay = SCT. Data for bar charts of proportional representation of cell populations were created using prop.table with margin = 2 and then visualized using ggplot2 (v3.3.5).

### Histology, immunohistochemistry, and *in situ* hybridization

Ferrets were euthanized as described previously, and nasal turbinates were fixed in 10% neutral-buffered formalin then embedded in paraffin and sectioned (9). Viral antigens and mRNAs were detected via immunohistochemistry (IHC) and *in situ* hybridization (ISH), respectively. IHC staining was performed using the Ventana Discovery Ultra Autostainer (Roche Ventana, Tucson, Arizona, USA) following the manufacturer’s instructions. Five micrometer formalin-fixed paraffin-embedded (FFPE) sections were initially heated for 4 min at 72°C and placed in EZ prep solution (950-102, Roche Ventana) for deparaffinization. Antigen retrieval was performed at 95°C in Cell Conditioning Solution 1 (950-124, Roche Ventana) for 56 min. A polyclonal primary goat polyclonal antibody (US Biological, Swampscott, MA) against A/USSR/90/1977 (H1N1) at 1:1,000 and a secondary biotinylated donkey anti-goat antibody (sc-2042; Santa Cruz Biotechnology, Santa Cruz, CA) at 1:200 on tissue sections. The DISCOVERY ChromoMap DAB detection kit (760-159, Roche Ventana) was used as the detection system. Tissue counterstaining was performed with Hematoxylin II solution (790-2208, Roche Ventana). To detect influenza viral and target mRNAs in FFPE tissues, *in situ* hybridization (ISH) was performed using the RNAscope 2.5 HD RED kit (Advanced Cell Diagnostics) according to the manufacturer’s instructions. Briefly, 40 ISH probes targeting influenza RNA and selected mRNA targets were designed and synthesized by Advanced Cell Diagnostics. Tissue sections were deparaffinized with xylene, underwent a series of ethanol washes and peroxidase blocking, and were then treated using kit-provided antigen retrieval buffer and proteinase. ISH signal was developed using a kit-provided pre-amplifier and amplifier conjugated to alkaline phosphatase and incubated with a fast red substrate solution for 10 minutes at room temperature. Sections were then counterstained with hematoxylin. All histologic sections were evaluated by a pathologist blinded to group assignments. Cell types in these sections were identifiable based on histologic features including their location and morphology (cell shape, arrangement, and nuclear and cytoplasmic features).

## RESULTS

### Responses to AIVs of different ATs in the ferret upper respiratory tract

We inoculated ferrets with subtype H1N1 AIVs isolated from wild birds during surveillance at Delaware Bay, DE, USA that showed different transmissibility in a ferret model (8, 9) (Table S1). The AIVs used here were A/ruddy turnstone/Delaware/300/2009 (H1N1) and A/ruddy turnstone/Delaware/AI09_256/2009 (H1N1) (DE300 and DE256, respectively), which showed AT, and A/shorebird/Delaware Bay/558/2006 (H1N1) and A/ruddy turnstone/Delaware/300/2009 (H1N1) with S213P introduced into the non-structural gene segment NS1 (DE558 and DE213, respectively), which did not show any evidence of AT (9). DE300 and DE213 were produced by rg, whereas DE256 and DE558 were wild-type viruses propagated from isolates. Our previous studies did not reveal any differences in viral replication kinetics *in vitro* or *in vivo,* and at 5 days post-inoculation (DPI), tissue pathology following inoculation of these viruses was similar throughout the respiratory tract with the exception of the nasal respiratory and olfactory neuroepithelia (9). Based on these findings, we focused on the nasal respiratory epithelium, including the olfactory neuroepithelium, at 2 DPI to study responses earlier in the course of infection.

**Fig 1:**
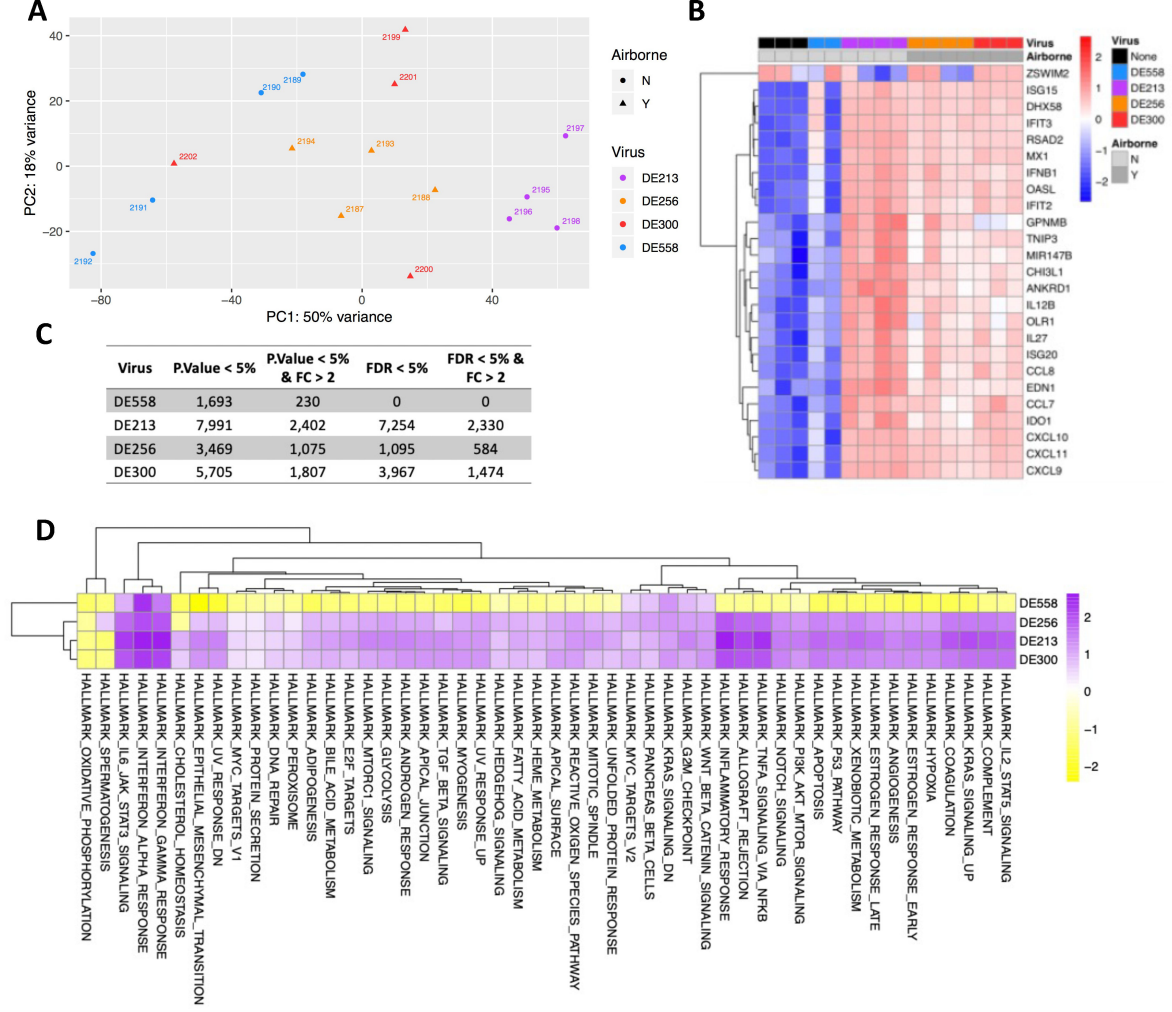
Ferret gene expression profile changes following viral inoculation. (**A**) Principal component analysis of the top 3,000 most variable features. (**B**) Z-score scaled log_2_ counts per million are shown for the top 50 most variable genes across all samples. (**C**) Table of the number of differentially expressed genes relative to no infection controls using various significant thresholds. (**D**) Heatmap of normalized enrichment scores from Hallmark gene set enrichment analysis.

We first studied gene expression at the tissue level using bulk RNA sequencing from cells obtained from the ferret nasal epithelium. At 2DPI, principal component analysis (PCA) of bulk RNA-seq data generally grouped profiles by infecting virus, demonstrating a virus-specific nature of the host response. The AT DE300 and DE256 showed similar expression profiles based on the top 3,000 most variable features, whereas the expression profiles associated with the not AT DE213 and DE558 were different (Fig. 1A). This is despite DE300 and DE213 differing by only a single amino acid. Compared with the expression profile associated with DE300, DE256, and DE213 inoculation, DE558 was the most dissimilar and clustered closer to uninoculated ferrets, which was also observed in the analysis of the top 50 most variable genes (Fig. 1B). This pattern was also evident in the number of differentially expressed genes in inoculated vs. uninoculated ferrets, in which DE558 had the least differentially expressed genes, followed by DE256, DE300, and DE213 (Fig. 1C). Therefore, encouragingly, virus transmission phenotype was a stronger driver of expression profile grouping than overall genetic similarity.

We next studied the gene expression differences between viruses. Of the top 50 differentially expressed genes, 49 were upregulated in ferrets inoculated with DE213, DE300, and DE256, with only one being downregulated (Fig. 1B). The downregulated gene was ZSWIM2, which has a known role in regulating Fas-, DR3-, and DR4-mediated apoptosis (24). ZSWIM2 was downregulated in three of four DE213-inoculated ferrets and two of four DE256-inoculated ferrets. However, it was not downregulated in DE300- or DE558-inoculated ferrets. The top 50 differentially expressed genes in DE300-, DE213-, and DE256-inoculated ferrets were all largely involved in the antiviral response, including MX1, a well-characterized host response gene against influenza viruses (25). Gene set enrichment pathway analysis (GSEA) revealed that of the 50 pathways in the analysis, all but eight were strongly upregulated in DE213-, DE256-, and DE300-inoculated ferrets compared with DE558-inoculated ferrets (Fig. 1D). Three immune pathways, the interferon alpha and gamma responses and the IL-6 JAK-STAT signaling pathway, were upregulated in all virus-inoculated ferrets. The oxidative phosphorylation pathway and the unrelated spermatogenesis pathway were downregulated in all inoculated ferrets relative to uninoculated ferrets, indicating that these may be common to responses to viral infections in the upper respiratory tract. Therefore, at the tissue level, the responses elicited by DE300, DE256, and DE213 were relatively similar compared with DE558.

### Role of NS1 in gene expression differences in the ferret nasal turbinate

**Fig 2:**
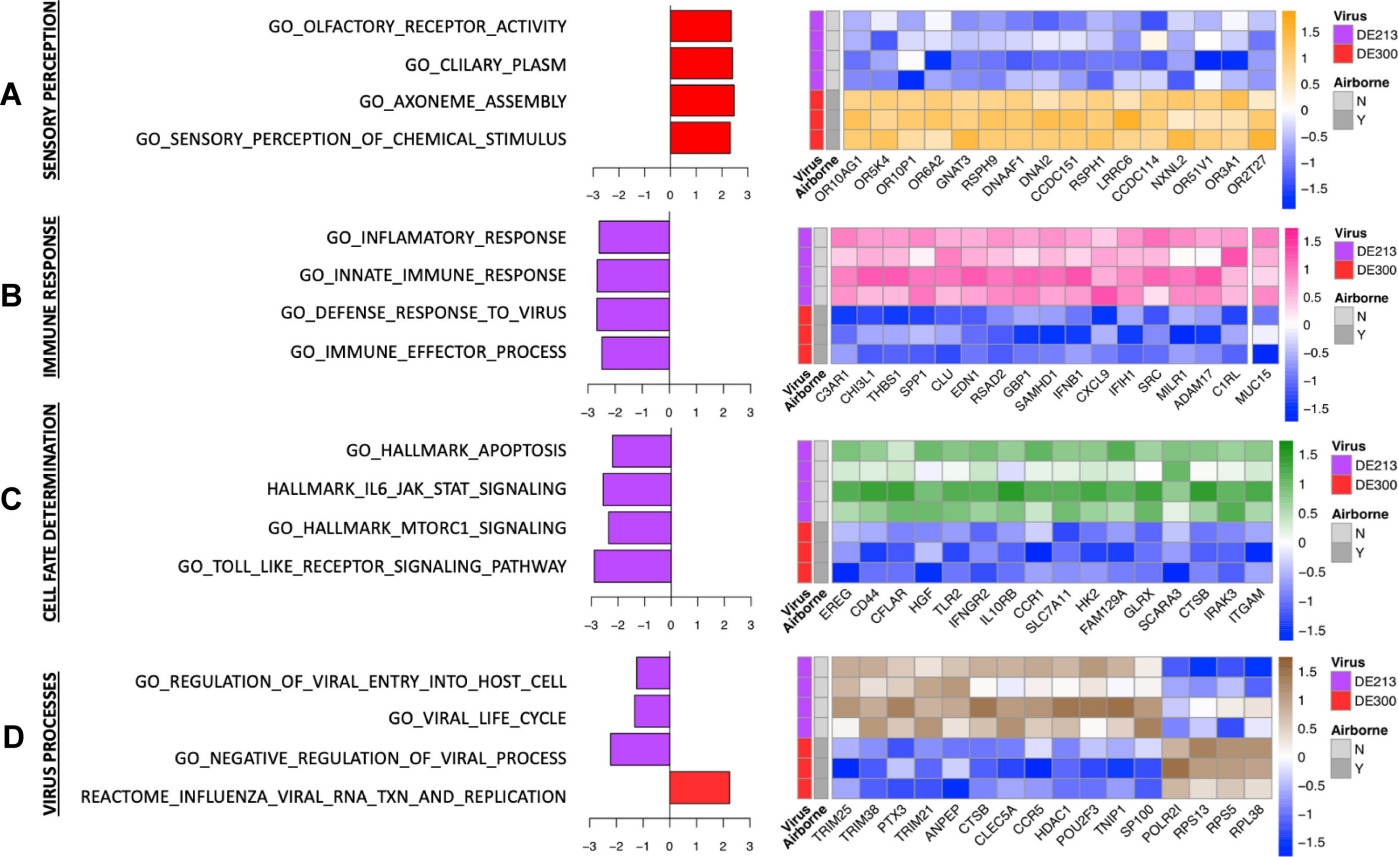
Pathway expression differences in ferrets following viral inoculation. Normalized enrichment score and z-scaled log_2_ counts per million for the top four enriched genes for each of the following pathways: (**A**) sensory perception, (**B**) immune response, (**C**) cell fate, and (**D**) biological processes associated with viral infections.

We next studied the responses to DE213 and DE300 to further dissect host responses to viruses of different AT phenotypes as these viruses were similar in terms of both the host responses elicited and their genomes but different in their AT phenotypes. A comparison of the host responses elicited by DE300 and DE213 revealed significant effects on several pathways. Pathways related to olfaction were upregulated in DE300-inoculated ferrets, these being olfactory receptor (OR) activity and pathways associated with the assembly and formation of the ciliary axoneme and basal body (Fig. 2A). ORs are a large family of G-protein coupled receptors that, along with cilia expressed on olfactory neurons, mediate the transduction of olfactory detection to the olfactory bulb (26, 27).

The innate immune response was also significantly affected, as innate immune pathways were upregulated in DE213-inoculated ferrets (Fig. 2B). MUC15, encoding the cell-surface mucin MUC15, which is a known immune mediator in the context of influenza virus infection, was also upregulated in DE213-inoculated ferrets (28) (Fig. 2B). Apoptosis pathways were also upregulated in DE213-inoculated ferrets (Fig. 2C). CD44, best characterized as a T-cell adhesion receptor and a mediator of apoptosis in T cells, was upregulated (29, 30). Caspase-8 and FADD Like Apoptosis Regulator (CFLAR), a regulator of CASP8-mediated apoptosis, and Hepatocyte Growth Factor (HGF) and Epiregulin (EREG), which have roles in wound healing in the respiratory epithelium, were downregulated in DE300-inoculated ferrets (31–33). Three Tripartite Motif (TRIM) genes were downregulated in DE300-inoculated ferrets; TRIM21, 25, and 38. TRIMs are involved in the regulation of several pathways that are important for the antiviral response, including the type-I interferon, ubiquitin, and NF-κB pathways (34). Pentraxin (PTX3), shown to interact with the influenza virus HA protein with inhibitory and neutralization activity, was also downregulated in DE300-inoculated ferrets (35, 36). Overall, these data indicate that downregulation of apoptosis pathways and innate immune signaling was associated with DE300 infection in the upper respiratory tract.

Genes that have been associated with the replication of viral RNA, namely POLR2I, which encodes a subunit of RNA polymerase II, and RPS13, RPS5, and RPL38, which encode ribosomal proteins, were upregulated in DE300-inoculated ferrets (Fig. 2D), suggesting that there may be differences in the viral transcription kinetics between DE300 and DE213, although this did not translate to differences in infectious viral titers in nasal washes. Two proteases involved in the expression or cleavage of the HA gene were also upregulated in DE300-inoculated ferrets; Alanyl (membrane), Aminopeptidase (ANPEP), and Cathepsin B (CTSB) (37, 38), as was C-Type Lectin Domain Family 5 Member A (CLEC5A), which can promote the production of proinflammatory cytokines and chemokines in myeloid cells in response to influenza virus infection (39). Chemokine receptor 5 (CCR5), histone deacetylase 1 (HDAC1), and TNFAIP3 Interacting Protein (TNIP1), important mediators of the inflammatory response in the respiratory tract, particularly via the regulation of neutrophils, were also downregulated in DE300-inoculated ferrets (40–43). Speckled Protein of 100 kDa (SP100) is an interferon-induced protein located in the nucleus that mediates early antiviral responses and was also downregulated in DE300 inoculated ferrets (44, 45). Collectively, these data demonstrate that a greater host antiviral signal was associated with DE213 infection in the upper respiratory tract.

### Single-cell RNA-seq of cell populations driving host responses to AIVs of differing AT in the ferret nasal turbinate

To identify cell populations contributing to observed bulk transcriptomic signatures of airborne transmissibility, we then performed single-cell gene expression (scGEX) analyses on cells isolated from ferret nasal turbinates 2DPI with DE558, DE256, DE300, or DE213 or mock inoculated with PBS (*n* = 4 ferrets per group). Single-cell libraries, excluding one library from DE256 that had poor quality control metrics after processing in CellRanger, were aggregated for analysis. After initial quality control processing steps, we included 114,363 single cells in 16 libraries, 33,740 from DE256 (three libraries), 25,783 from DE558 (four libraries), 18,336 from DE213 (four libraries), 28,445 from DE300 (four libraries), and 8,049 cells from mock-infected (one library) for formal analyses.

**Fig 3:**
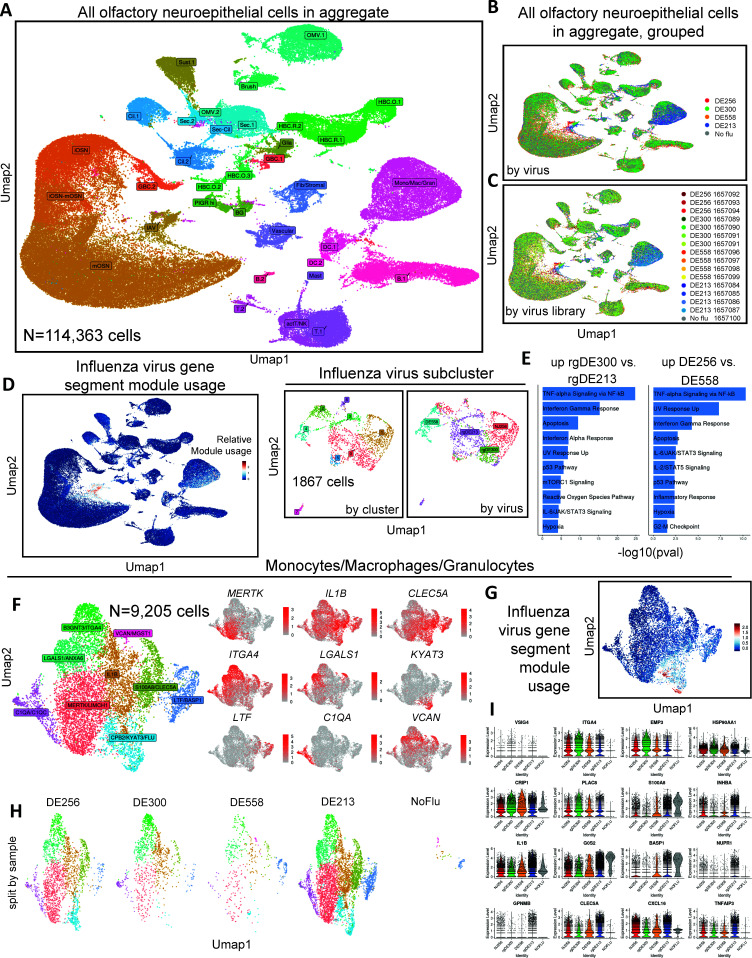
Single-cell analysis of ferret olfactory neuroepithelium demonstrates specific differences in host transcriptional responses. Uniform manifold approximation and projection (UMAP) of all cells from the olfactory neuroepithelium labeled by (**A**) putative cell type, (**B**) virus, and (**C**) sample. (**D**) UMAP following subsetting and reclustering using SCTransform with influenza virus gene expression as a variable. (**E**) Bar plots of pathways (MsigDB Hallmark) enriched when comparing rgDE300 and rgDE213 cells, and, DE256 and DE558 cells. (**F and G**) UMAP following subclustering of monocyte/macrophage/granulocyte populations using SCTransform, with influenza virus gene expression as a variable, showing nine transcriptionally distinct granulocyte, monocyte, and macrophage populations. (**H**) UMAP of cells following subclustering of monocyte/macrophage/granulocyte populations split by a virus. (**I**) Violin plots grouped by infecting virus of interferon-stimulated gene (ISG) module usage (46) among IAV infected cells, denoted “IAV” in (**A**), with black bars representing median module usage and gene expression level.

Using previously published literature as a guide (22, 23), we annotated clusters in the aggregate data set (Fig. 3A and Fig. S1). These included populations of immune cells, including mononuclear phagocytes (*CD14*, *CSF3R*, and *TIFAB*), B cells (*CD19*) and T cells (*CD3E*), and non-immune cells, including olfactory neurons along the developmental trajectory from the globose basal cell (*NEUROD1*) to mature olfactory neurons (*GNG13*), sustentacular cells (*ERMN*), glial cells (*ERMN* and *GPM6B*), olfactory and respiratory horizontal basal cells (O/RHBCs, *TP63* and *KRT5*), olfactory microvillar cells (OMVs; *HEPACAM2*), brush cells (*CHAT* and *TRPM5*), secretory cells (*SCGB1A1*), ciliated cells (*PIFO* and *CCDC153*), fibroblasts (*PDGFRA* and *COL1A1*), and vascular cells (*CDH5*) (Fig. S2A). One cluster in particular that expressed both neuronal and respiratory secretory cell markers was enriched in influenza virus gene segment expression and was named IAV in the annotation (Fig. 3B). We observed lower expression of multiple gene segments from each virus in other cell clusters, including the monocyte/macrophage/granulocyte clusters and ciliated epithelial cells (Fig. S2B). Grouping cells by sample and by virus demonstrated overlapping clustering largely irrespective of library or virus with two notable exceptions, the IAV and monocytes/macrophages/granulocytes clusters (Fig. 3C and Fig. S2C).

Given the observed differences in clustering by the virus within the IAV cell population, we subsetted and reclustered these cells using SCTransform with IAV gene expression as a variable to regress for additional analyses. The IAV subset included 1,867 cells, 705 from DE256, 252 from DE300, 234 from DE558, 676 from DE213, and zero cells from uninoculated ferrets (Fig. 3D, left). Visualization revealed that these cells largely still clustered according to the infecting virus (Fig. 3D, right) and appeared to be respiratory epithelial cells expressing secretory cell-associated proteins (*SCGB1A1, LYPD2*) and, to a lesser extent, mature olfactory neurons (*GNG13*) (Fig. S2D). Of note, there was little proportional representation of cells from DE558-infected ferrets in clusters expressing secretory transcripts and little proportional representation of cells from DE300-infected ferrets in clusters expressing neuronal markers (Fig. S2E).

Comparisons of DE300 to DE213 via pathway enrichment analysis (MsigHallmark Database 2020) revealed the top upregulated pathways to be TNF signaling, interferon-gamma and -alpha responses, apoptosis, and the UV response (Fig. 3E, left). The same analysis comparing DE256 to DE558 revealed upregulation of similar pathways, except for interferon-alpha responses (Fig. 3E, right). Interestingly, TNF signaling, interferon signaling, and apoptosis pathways were not upregulated in DE256 relative to DE213 (Fig. S2F). We next performed comparisons of DE300 vs. DE213 and DE256 vs. DE558 using DGE testing on corrected, log-normalized counts to identify a list of significantly upregulated genes in each comparison (Fig. S2G). These significantly upregulated genes included those associated with Nuclear Factor Kappa Beta (NF-κB) signaling, cellular stress (*GADD45β*), and secretory cell-associated transcripts (*BPIFA1* and *SCGB1A1*). However, consistent with pathway analyses, the expressions of many genes between DE256 and DE213 were not different, except for *BPIFA1* and *SCGB1A1*. Finally, we took an interest in *NUP153*, *RNF213*, and *TRIM22,* which were differentially upregulated between DE300 and DE213 and have previously been implicated in host-influenza virus interactions (Fig. S2H). Taken together, these analyses suggest that cellular genotoxic stress (*GADD45β*), NF-κB, interferon signaling, and apoptosis were important differentiating features of DE300 relative to DE213 and DE256 relative to DE558.

We next studied cells of the immune system by subclustering monocyte/macrophage/granulocyte populations for further analysis using SCTransform, with IAV gene segment expression included as a variable to regress. This subpopulation included cells co-expressing non-myeloid cell-associated transcripts, potentially suggestive of doublets (biological, technical, or otherwise) (Fig. S2I). To focus specifically on myeloid cell populations, we removed these contaminating cell populations for further reclustering. This population included 9,205 cells in total, 2,276 from DE256-infected ferrets, 1,104 from DE300-infected ferrets, 487 from DE558-infected ferrets, 5,247 from DE213-infected ferrets, and 91 from mock-inoculated ferrets. In this population, we identified nine transcriptionally distinct granulocyte, monocyte, and macrophage populations. We labeled these according to the transcripts significantly enriched within each population, which included IAV gene segments within the *CPB2/KYAT3/FLU* cluster (Fig. 3F and G). We noted that the presence of IAV gene segments in these phagocytic cells may have been due to the phagocytosis of other infected cells.

In keeping with findings from previous analyses (Fig. S2A), there were more monocyte/macrophage/granulocyte cells originating from ferrets inoculated with DE213 relative to other IAVs (Fig. S2J). Comparing proportional enrichment of individual cell populations between infecting IAVs demonstrated that a higher proportion of cells originating from ferrets inoculated with DE213 and DE256 clustered into the *MERTK* and *CPB2/KYAT3/FLU* populations compared with DE300, where a higher proportion of cells clustered into *IL1B* and *ITGA4* populations (Fig. 3H and Fig. S2K). We finally performed differential gene expression analysis to identify individual transcripts enriched in DE300 relative to DE213 and vice-versa (Fig. 3I and Fig. S2L). Several of these, including *GPNMB*, *OLR1*, *CHI3L1*, *TNIP3*, and *CLEC5A,* were identified as upregulated in DE213 in bulk transcriptomic analyses. Overall, these data reveal that innate immune responses were affected by AIVs of different AT phenotypes.

### Pathology in the ferret upper respiratory tract associated with AIV AT

**Fig 4:**
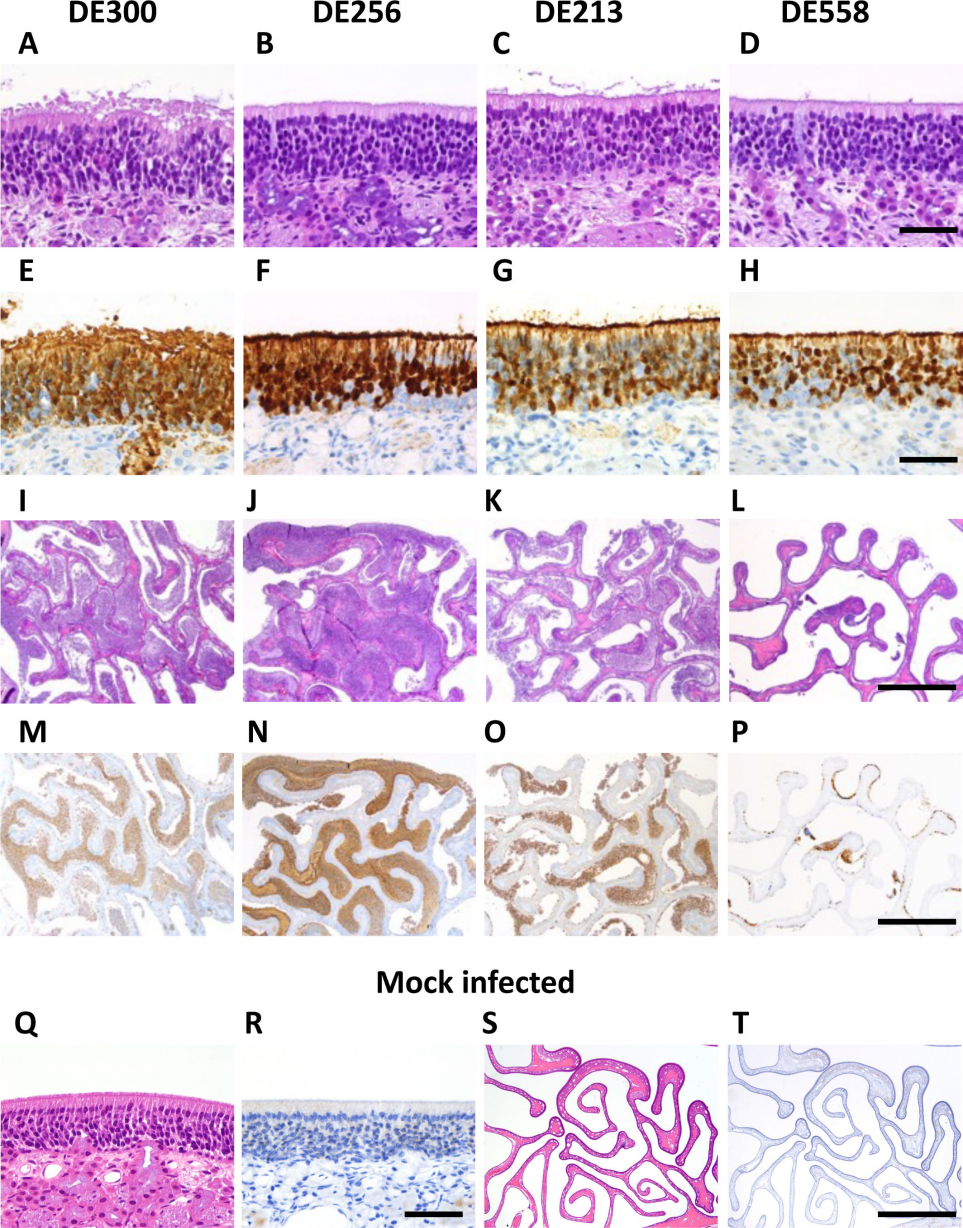
Differences in tissue pathology and exudate in the nasal epithelium of virus-inoculated ferrets. In the nasal epithelium of ferrets inoculated with DE300 and DE256, lesions were indicative of pyroptosis and necroptosis, leading to an almost complete loss of ciliated respiratory epithelium (**A, B, E, and F**). An exudate consisting of abundant serum and cytoplasmic blebs was also present (**I, J, M, and N**). In the nasal epithelium of ferrets inoculated with DE213 and DE558, cytolysis was the predominant cause of cell death (**C, D, G, and H**). The nasal exudates also appeared to be more densely cellular and less intensely stained for viral antigens (**K, L, O, and P**). Mock-infected ferrets are shown in Q–T. Scale bars in D, H, and R = 50 µm. Scale bars in L, P, and T = 1 mm.

We next studied the pathology in the olfactory neuroepithelium of ferrets inoculated with DE300, DE256, DE558, or DE213 at 2DPI as our previous studies performed at 5DPI revealed that the only observable pathological differences between AIVs in the respiratory tract were observed at the olfactory neuroepithelium. DE300 and DE256 were cytolytic and led to an almost complete loss of ciliated respiratory epithelium and the presence of an exudate containing abundant serum and cytoplasmic blebs (Fig. 4A, B, E, F, I, J, M, N and Q through T). Lesions observed in these tissues of ferrets inoculated with DE300 and DE256 were indicative of pyroptosis and necroptosis being the predominant mechanism of cell death. The nasal exudates in these animals were characterized by abundant cytoplasmic blebs and serum. In contrast, in ferrets inoculated with DE558 or DE213, there were still numerous surviving, but often infected, ciliated respiratory epithelial cells present, and apoptosis appeared to be the primary phenotype of cell death, as observed in the gene expression profiles. Cytoplasmic blebs and serum were rarely present, and the nasal exudates appeared to be more densely cellular and less intensely stained for viral antigens (Fig. 4C, D, G, H, K, L, O, P and Q through T). These features may underlie the production of larger respiratory particles containing less virus. Taken together, these findings suggest that cytolytic infections may be associated with the production of infectious respiratory particles.

We next used ISH to study genes identified in our transcriptomic studies as differentially expressed in ferrets inoculated with AIVs of different AT phenotypes. Of these, we selected genes associated with host responses to infection in the respiratory tract. Bactericidal/permeability-increasing fold containing family A member 1 (BPIFA1) is a secreted lipid binding protein involved in the regulation of surfactant and airway surface liquid homeostasis that is associated with protection from bacterial and viral infections of the upper respiratory tract, including influenza virus (47). C-X-C Motif Chemokine Ligand 10 (CXCL10) is a chemokine-induced in response to inflammation or infection that plays an important role in mediating lung injury (48, 49). 2'−5′-Oligoadenylate Synthetase 2 (OAS2) is an antiviral enzyme activated by the innate immune response that catalyzes the synthesis of 2′−5′-oligoadenylate for RNase L activation and subsequent interferon production among other responses (50). Interferon-stimulated gene 15 (ISG15) is an abundant interferon-stimulated gene known to have several roles in response to viral infections (51). Clusterin (CLU) has regulatory roles in cell cycle and survival and has been shown to interact with influenza virus nucleoprotein (52). Cytidine/Uridine Monophosphate Kinase 2 (CMPK2) is an activator of the NLRP3 inflammasome that is differentially expressed in response to SARS-CoV-2, influenza virus, rhinovirus, and respiratory syncytial virus infection (53). Beta-2-microglobulin (B2M) is a component of the major histocompatibility complex class I and is involved in the pathogenesis of respiratory diseases (54, 55). CD81 is a tetraspanin involved in B-cell signaling that also has roles in the entry and the budding of influenza virus (56). Cytochrome P450 2F5 (ferret LOC101675002) is a member of a family of monooxygenases with roles in lung injury and oxidative stress (57, 58). Finally, speckled protein 100 kDa (SP100) is an interferon-stimulated gene that is an important mediator of innate immunity and is involved in antiviral responses (59, 60).

**Fig 5:**
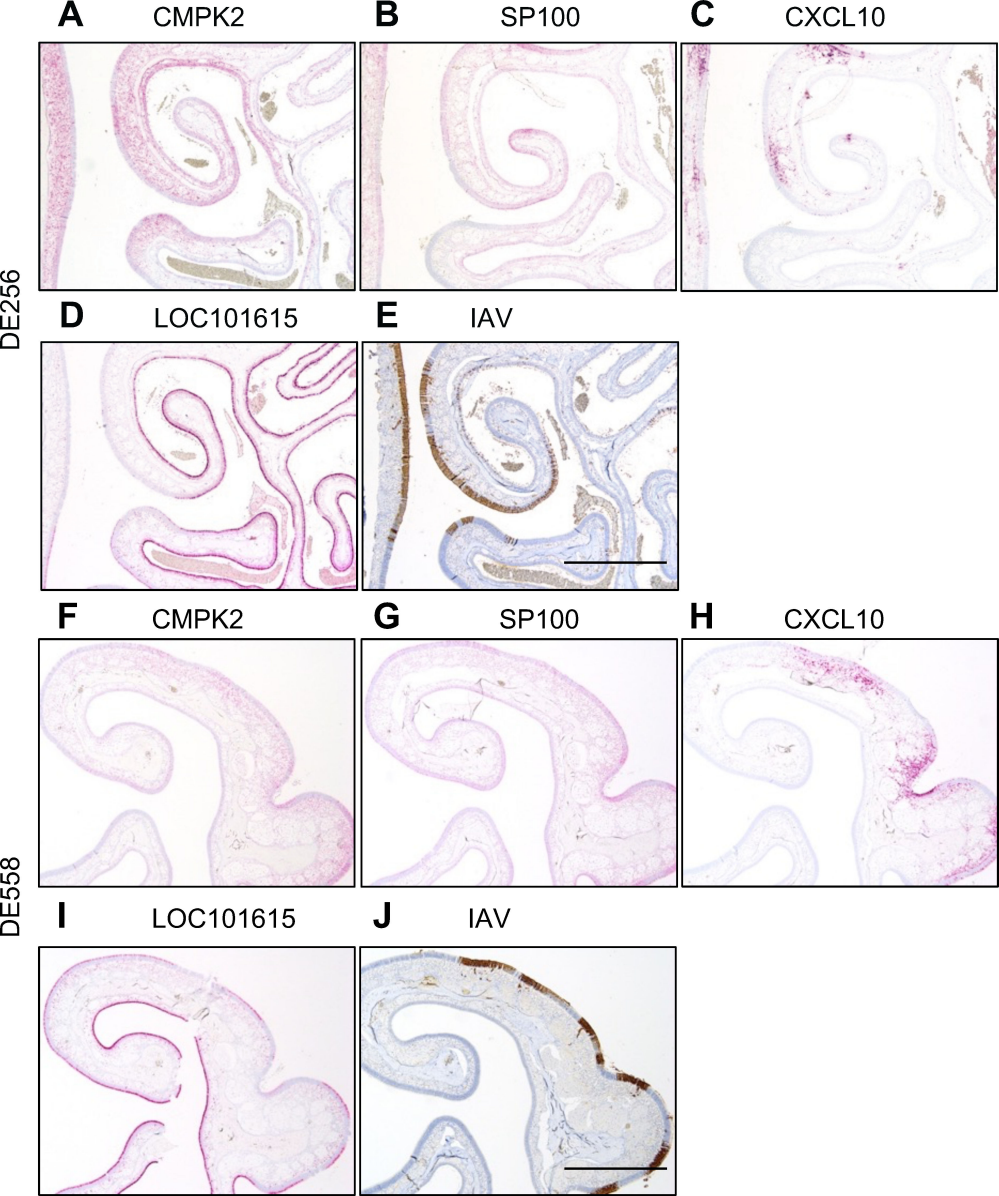
Gene expression in ferret nasal epithelium of virus-inoculated ferrets by *in situ* hybridization. Nasal turbinates of ferrets inoculated with DE256 (**A–E**) or DE558 (**G–J**) were probed for the expression of CMPK2 (A&F), SP100 (B&G), CXCL10 (C&H), LOC101675002 (D&I), and influenza A virus (IAV, E&J). Scale bars = 1 mm.

We performed ISH on the nasal tissues of ferrets inoculated with DE256 and DE558 to compare gene expression in ferrets inoculated with these AIVs. *CMPK2* was detected as multifocal labeling in the nose of DE558-inoculated ferrets and as diffuse and extensive labeling in DE256-inoculated ferrets (Fig. 5A and F; Table S2). Diffuse and extensive labeling of *SP100* was evident in the respiratory epithelium and olfactory neuroepithelium of DE256-inoculated ferrets, respectively, whereas multifocal labeling of the respiratory and olfactory epithelia was evident in DE558-inoculated ferrets (Fig. 5B and G, Table S2). Strong labeling of *CXCL10* was evident in the respiratory epithelium and associated submucosa of DE256-inoculated ferrets, whereas patchy labeling of turbinates was evident in DE558-inoculated ferrets (Fig. 5C and H; Table S2). Labeling of *LOC101067500*, *BPIFA1*, *CLU,* and *ISG15* was similar between ferrets inoculated with DE256 and DE558 (Fig. 5D and I; Table S2). *B2M* labeling was inconsistent in DE256-inoculated ferrets and was multifocal in the respiratory and olfactory epithelia of DE558-inoculated ferrets (Table S2). Labeling of *CD81* was inconsistent between ferrets inoculated with different AIVs in the nasal epithelium, and *OAS2* labeling was not detected (Table S2). We also observed that influenza staining was more focused on the debris present in the nasal turbinates in ferrets inoculated with DE256, whereas it was more focused on the epithelium of these tissues in ferrets inoculated with DE558 (Fig. 5E and J; Table S2). Overall, of the targets studied, we observed different labeling patterns of *CMPK2*, *SP100,* and *CXCL10* between ferrets inoculated with DE256 or DE558.

### Differences in AT were not associated with changes in viral diversity

Studies of subtype H7N9 AIVs infection in ferrets revealed the existence of a “genetic bottleneck” that limited viral adaptability to a mammalian host by limiting genetic diversity in that host (61). As such, we sought to determine if there were any differences in viral diversity between AIVs of different AT phenotypes. Overall, there did not appear to be any differences in the diversity of viral populations studied in ferret nasal washes at 2DPI. As viral diversity can be more limited in viral stocks produced by rg, here, we had two pairs of viruses of different AT phenotypes, a pair of rg viruses (rgDE300 and rgDE213) and a pair of wild-type viruses (DE256 and DE558). Shannon entropy analysis revealed a hot spot of complexity in the HA gene, in nucleotides 216–218, located in the globular head domain. However, the entropy at this position was similar between stock viruses and viruses in ferret nasal washes. Furthermore, there were no significant differences in entropy at this position between viruses in ferret nasal washes (Fig. S3A and 3B). Large potential deletions were present in the polymerase genes of all viruses, which were indicative of the presence of a population of defective interfering (DI) particles that are formed containing incomplete viral genomes (62, 63). There was little evidence of DI particles in DE300 (Fig. S3C), whereas coverage at the ends of the polymerase genes was greater in DE213, particularly in the PA gene segment, which could indicate increased DI particles in the DE213 population, although the sample size of DE213 was limited (*n* = 2) (Fig. S3D). Indications of DI particles were stronger in the DE256 and DE558 populations (Fig. S3E and F), but no relatively large differences were observed between viruses. It should also be noted that, as DE300 and DE213 were generated by reverse genetics, it would be expected that population diversity in these viruses would be less compared with viruses propagated from isolates. Overall, these data indicate that viral population diversity was not a driving factor in host responses to these viruses.

## DISCUSSION

Human cases of AIV infection have generally originated from exposure to infected poultry or swine rather than with wild birds. This is likely simply due to the closer proximity of humans to poultry and swine as a part of farming practices, whereas direct contact with wild birds is much rarer. However, the capacity for mammalian infection, replication, and even severe disease in the murine model appears to exist in AIVs maintained in avian reservoirs that presumably have not undergone prior adaptation to a mammalian host. These viruses can also show a capacity for infection and shedding in the ferret model, with contact transmission and, in some cases, AT, evident. Considering this and the importance of this wild bird reservoir in the maintenance of IAVs in the environment, we endeavored to gain insights into the host-virus interactions in the upper respiratory tract that may be determinants of AT. We used a well-established ferret model of human influenza virus transmission to study host responses at the determinant site of airborne transmission, the upper respiratory tract. We took advantage of the relatively new annotation of the ferret genome to inform our analysis of bulk and single-cell transcriptomic studies of the ferret nasal epithelium.

Our previous studies of these pathogeneses and transmissions of these AIVs were conducted in ferrets at 5 DPI (9). At this time point, pathology studies of the entire respiratory tract revealed that the olfactory neuroepithelium was the only location with different pathology between AIVs of different AT phenotypes. Furthermore, the pathology of the URT epithelium was extensive, suggesting that an earlier time point may be more sensitive to differences between AT phenotypes. Furthermore, nasal wash viral titers were detected in airborne contact ferrets as early as three days after exposure to inoculated ferrets, indicating that AT events occurred before this. As nasal wash viral titers were detected as early as 2 DPI in inoculated ferrets, we chose this time point for the studies described here to gain insights into events earlier during the course of infection that may have influenced AT phenotypes.

Responses to infection with AIVs in the upper respiratory tract were characterized by upregulation of the IFN-α, IFN-γ, and the IL-6 JAK-STAT pathways. Type I IFNs, including IFN-α, are activated within hours of IAV infection and induce antiviral states through signaling via type 1 IFN-α/β receptors (IFNARs) and the JAK-STAT pathway in proximal, uninfected cells. This leads to the expression of interferon-stimulated genes (ISGs) and the induction of an antiviral state in uninfected cells to limit viral spread. There was also a downregulation of the oxidative phosphorylation pathway. Influenza virus infection is associated with disruption of mitochondria and thus cellular metabolism, leading to increased oxidative stress associated with the increased reactive oxygen species that, in turn, can lead to increased inflammation and cell death. Mitochondria are also involved in the induction of antiviral immunity through the OXPHOS activity (64, 65). The downregulation of this pathway by AIVs here could represent a host antiviral response suppressed by these viruses. Further studies into this host-virus interaction, particularly to determine if this is particular to AIVs compared with seasonal strains, would be interesting. It should also be noted that gene expression differences were not confirmed by qPCR. Furthermore, this study used 3-month-old ferrets, which are considered juveniles. As age is a determinant of susceptibility to influenza, it would be interesting to determine if similar responses are observed in adult ferrets of 6 to 12 months old (66).

We also studied rgDE300 and rgDE213, which differed only at amino acid 213 in NS1. S213P in rgDE300 reduced the airborne transmissibility of this virus in a ferret model. NS1 is an extensively studied IAV protein that appears to be the main antagonist of the host antiviral response, impacting pathways involved in innate immunity, cell fate, and pathogen sensing to engineer an environment more conducive to viral replication. NS1 S213P resulted in reduced expression of host antiviral signaling pathways, such as the inflammatory and innate immune response and cell death pathways. The importance of these pathways, as observed in the bulk RNA-seq analysis, was also evident in single-cell RNA-seq performed on the “IAV” cell cluster, whereby the three top differentially expressed pathways between rgDE300 and rgDE213, being upregulated in rgDE300, were TNF-α signaling via NF-κB, IFN-γ response, and apoptosis pathways. However, interestingly, the upregulation of these pathways was the opposite in the single-cell RNA-seq data set compared with the bulk RNA-seq data set, in that upregulation of these pathways was evident in association with DE300 or DE256 compared with DE213 or DE558. These data indicate that host responses elicited at the site of infection are different compared with those at the tissue level, which could potentially be influenced by host responses at uninfected sites.

The data obtained from single-cell RNA-seq appeared to be reflected by what was observed microscopically in the pathology studies, whereby greater damage to the epithelium appeared in ferrets inoculated with rgDE300 compared with rgDE213. According to these results, mechanisms of cell death in ferret nasal epithelium inoculated with DE300 or DE256 appeared to be pyroptosis and necroptosis, both inflammatory modes of cell death, whereas apoptosis was more associated with DE558 or DE213. This could also potentially explain the increased expression of inflammatory signaling pathways observed in these ferrets. It is possible that these differing mechanisms of epithelial damage and cell death may be a determinant of transmissibility as infection with AIVs associated with AT was linked to stronger IAV antigen staining of debris and the presence of serum and cytoplasmic blebs not evident in the debris associated with AIVs not associated with AT. Considering these differences, that there is an association between them and the transmissibility of these viruses, and their location in the upper respiratory tract, we hypothesize that this debris could be the source material for virus-laden particles that mediate/determine the transmissibility of these viruses. Further study is needed to determine and characterize any potential changes in expelled particles from ferrets inoculated with these viruses. IHC studies revealed different labeling of CMPK2, SP100, and CXCL10 in the nasal epithelia of ferrets inoculated with DE256 or DE558. As described above, these genes encode proteins with known roles in the innate immune response to viral infections of the respiratory tract, supporting the transcriptomic data and the importance of innate immunity as a determinant of AIV transmissibility in the upper respiratory tract.

In summary, our study has revealed differences in the mammalian host responses to infection with AIVs of differing AT in the upper respiratory tract, the determinant anatomical site of influenza virus transmission. Cellular genotoxic stress, NF-kB, interferon, and cell fate pathways differentiated responses to AIVs of different AT. Hallmarks of AT AIV infection were the presence of AIV antigen-containing exudate and debris in the respiratory spaces of the nasal epithelium and differential expression of *CMPK2*, *SP100,* and *CXCL10* in infected epithelia. Overall, our study has revealed host responses underlying the AT potential of AIVs in mammals.

## Data Availability

RNA sequencing data are available from the Gene Expression Omnibus. Bulk RNA sequencing data are available under accession number GSE296526, and single cell RNA sequencing data are available under accession number GSE298058.

## SUPPLEMENTAL MATERIAL

Supplemental material jvi.00647-25-s0001.pdf Figures S1 to S3; Tables S1 and S2.

ASM does not own the copyrights to Supplemental Material that may be linked to, or accessed through, an article. The authors have granted ASM a non-exclusive, world-wide license to publish the Supplemental Material files. Please contact the corresponding author directly for reuse.

## References

[1] Koçer ZA, Krauss S, Stallknecht DE, Rehg JE, Webster RG. 2012. The potential of avian H1N1 influenza A viruses to replicate and cause disease in mammalian models. PLoS One 7:e41609. doi:10.1371/journal.pone.0041609 22848544 PMC3404991

[2] Zanin M, Koçer ZA, Poulson RL, Gabbard JD, Howerth EW, Jones CA, Friedman K, Seiler J, Danner A, Kercher L, McBride R, Paulson JC, Wentworth DE, Krauss S, Tompkins SM, Stallknecht DE, Webster RG. 2017. Potential for low-pathogenic avian H7 influenza A viruses to replicate and cause disease in a mammalian model. J Virol 91:e01934-16. doi:10.1128/JVI.01934-16 27852855 PMC5244340

[3] Kim EH, Kim YL, Kim SM, Yu KM, Casel MAB, Jang SG, Pascua PNQ, Webby RJ, Choi YK. 2021. Pathogenic assessment of avian influenza viruses in migratory birds. Emerg Microbes Infect 10:565–577. doi:10.1080/22221751.2021.1899769 33666526 PMC8018353

[4] Wang Y, Wang M, Zhang H, Zhao C, Zhang Y, He G, Deng G, Cui P, Li Y, Liu W, Shen J, Sun X, Wang W, Zeng X, Li Y, Chu D, Peng P, Guo J, Chen H, Li X. 2022. Emergence, evolution, and biological characteristics of H10N4 and H10N8 avian influenza viruses in migratory wild birds detected in Eastern China in 2020. Microbiol Spectr 10:e0080722. doi:10.1128/spectrum.00807-22 35389243 PMC9045299

[5] Yao Z, Zheng H, Xiong J, Ma L, Gui R, Zhu G, Li Y, Yang G, Chen G, Zhang J, Chen Q. 2022. Genetic and pathogenic characterization of avian influenza virus in migratory birds between 2015 and 2019 in Central China. Microbiol Spectr 10:e0165222. doi:10.1128/spectrum.01652-22 35862978 PMC9431584

[6] Lv X, Tian J, Li X, Bai X, Li Y, Li M, An Q, Song X, Xu Y, Sun H, et al.. 2023. H10Nx avian influenza viruses detected in wild birds in China pose potential threat to mammals. One Health 16:100515. doi:10.1016/j.onehlt.2023.100515 37363234 PMC10288057

[7] Kayed AE, Kutkat O, Kandeil A, Moatasim Y, El Taweel A, El Sayes M, El-Shesheny R, Aboulhoda BE, Abdeltawab NF, Kayali G, Ali MA, Ramadan MA. 2023. Comparative pathogenic potential of avian influenza H7N3 viruses isolated from wild birds in Egypt and their sensitivity to commercial antiviral drugs. Arch Virol 168:82. doi:10.1007/s00705-022-05646-w 36757481 PMC9909137

[8] Koçer ZA, Krauss S, Zanin M, Danner A, Gulati S, Jones JC, Friedman K, Graham A, Forrest H, Seiler J, Air GM, Webster RG. 2015. Possible basis for the emergence of H1N1 viruses with pandemic potential from avian hosts. Emerg Microbes Infect 4:e40. doi:10.1038/emi.2015.40 26251829 PMC4522614

[9] Zanin M, Wong SS, Barman S, Kaewborisuth C, Vogel P, Rubrum A, Darnell D, Marinova-Petkova A, Krauss S, Webby RJ, Webster RG. 2017. Molecular basis of mammalian transmissibility of avian H1N1 influenza viruses and their pandemic potential. Proc Natl Acad Sci USA 114:11217–11222. doi:10.1073/pnas.1713974114 28874549 PMC5651783

[10] Xie C, Su W, Sia SF, Choy KT, Morrell S, Zhou J, Peiris M, Bloom JD, Yen HL. 2022. A(H1N1)pdm09 influenza viruses replicating in ferret upper or lower respiratory tract differed in onward transmission potential by air. J Infect Dis 225:65–74. doi:10.1093/infdis/jiab286 34036370 PMC8730494

[11] Richard M, van den Brand JMA, Bestebroer TM, Lexmond P, de Meulder D, Fouchier RAM, Lowen AC, Herfst S. 2020. Influenza A viruses are transmitted via the air from the nasal respiratory epithelium of ferrets. Nat Commun 11:766. doi:10.1038/s41467-020-14626-0 32034144 PMC7005743

[12] Poulson R, Carter D, Beville S, Niles L, Dey A, Minton C, McKenzie P, Krauss S, Webby R, Webster R, Stallknecht DE. 2020. Influenza A viruses in ruddy turnstones (Arenaria interpres); connecting wintering and migratory sites with an ecological hotspot at Delaware Bay. Viruses 12:1205. doi:10.3390/v12111205 33105913 PMC7690596

[13] Andrews S. 2017. FastQC: a quality control tool for high throughput sequence data. Available from: http://www.bioinformatics.babraham.ac.uk/projects/fastqc

[14] Dobin A, Davis CA, Schlesinger F, Drenkow J, Zaleski C, Jha S, Batut P, Chaisson M, Gingeras TR. 2013. STAR: ultrafast universal RNA-seq aligner. Bioinformatics 29:15–21. doi:10.1093/bioinformatics/bts635 23104886 PMC3530905

[15] Li H, Durbin R. 2009. Fast and accurate short read alignment with Burrows–Wheeler transform. Bioinformatics 25:1754–1760. doi:10.1093/bioinformatics/btp324 19451168 PMC2705234

[16] Anders S, Pyl PT, Huber W. 2015. HTSeq--a Python framework to work with high-throughput sequencing data. Bioinformatics 31:166–169. doi:10.1093/bioinformatics/btu638 25260700 PMC4287950

[17] Law CW, Chen Y, Shi W, Smyth GK. 2014. voom: Precision weights unlock linear model analysis tools for RNA-seq read counts. Genome Biol 15:R29. doi:10.1186/gb-2014-15-2-r29 24485249 PMC4053721

[18] Liberzon A, Subramanian A, Pinchback R, Thorvaldsdóttir H, Tamayo P, Mesirov JP. 2011. Molecular signatures database (MSigDB) 3.0. Bioinformatics 27:1739–1740. doi:10.1093/bioinformatics/btr260 21546393 PMC3106198

[19] Subramanian A, Tamayo P, Mootha VK, Mukherjee S, Ebert BL, Gillette MA, Paulovich A, Pomeroy SL, Golub TR, Lander ES, Mesirov JP. 2005. Gene set enrichment analysis: a knowledge-based approach for interpreting genome-wide expression profiles. Proc Natl Acad Sci USA 102:15545–15550. doi:10.1073/pnas.0506580102 16199517 PMC1239896

[20] Piper C, Hainstock E, Yin-Yuan C, Chen Y, Khatun A, Kasmani MY, Evans J, Miller JA, Gorski J, Cui W, Drobyski WR. 2022. Single-cell immune profiling reveals a developmentally distinct CD4+ GM-CSF+ T-cell lineage that induces GI tract GVHD. Blood Adv 6:2791–2804. doi:10.1182/bloodadvances.2021006084 35015822 PMC9092418

[21] Hafemeister C, Satija R. 2019. Normalization and variance stabilization of single-cell RNA-seq data using regularized negative binomial regression. Genome Biol 20:296. doi:10.1186/s13059-019-1874-1 31870423 PMC6927181

[22] Durante MA, Kurtenbach S, Sargi ZB, Harbour JW, Choi R, Kurtenbach S, Goss GM, Matsunami H, Goldstein BJ. 2020. Single-cell analysis of olfactory neurogenesis and differentiation in adult humans. Nat Neurosci 23:323–326. doi:10.1038/s41593-020-0587-9 32066986 PMC7065961

[23] Fodoulian L, Tuberosa J, Rossier D, Boillat M, Kan C, Pauli V, Egervari K, Lobrinus JA, Landis BN, Carleton A, Rodriguez I. 2020. SARS-CoV-2 receptors and entry genes are expressed in the human olfactory neuroepithelium and brain. iScience 23:101839. doi:10.1016/j.isci.2020.101839 33251489 PMC7685946

[24] Nishito Y, Hasegawa M, Inohara N, Núñez G. 2006. MEX is a testis-specific E3 ubiquitin ligase that promotes death receptor-induced apoptosis. Biochem J 396:411–417. doi:10.1042/BJ20051814 16522193 PMC1482824

[25] Haller O, Kochs G. 2020. Mx genes: host determinants controlling influenza virus infection and trans-species transmission. Hum Genet 139:695–705. doi:10.1007/s00439-019-02092-8 31773252 PMC7087808

[26] Buck L, Axel R. 1991. A novel multigene family may encode odorant receptors: a molecular basis for odor recognition. Cell 65:175–187. doi:10.1016/0092-8674(91)90418-x 1840504

[27] Jenkins PM, McEwen DP, Martens JR. 2009. Olfactory cilia: linking sensory cilia function and human disease. Chem Senses 34:451–464. doi:10.1093/chemse/bjp020 19406873 PMC2682445

[28] Chen ZG, Wang ZN, Yan Y, Liu J, He TT, Thong KT, Ong YK, Chow VTK, Tan KS, Wang DY. 2019. Upregulation of cell-surface mucin MUC15 in human nasal epithelial cells upon influenza A virus infection. BMC Infect Dis 19:622. doi:10.1186/s12879-019-4213-y 31307416 PMC6631914

[29] Baaten BJ, Li CR, Bradley LM. 2010. Multifaceted regulation of T cells by CD44. Commun Integr Biol 3:508–512. doi:10.4161/cib.3.6.13495 21331226 PMC3038050

[30] DeGrendele HC, Estess P, Siegelman MH. 1997. Requirement for CD44 in activated T cell extravasation into an inflammatory site. Science 278:672–675. doi:10.1126/science.278.5338.672 9381175

[31] Narasaraju T, Ng HH, Phoon MC, Chow VTK. 2010. MCP-1 antibody treatment enhances damage and impedes repair of the alveolar epithelium in influenza pneumonitis. Am J Respir Cell Mol Biol 42:732–743. doi:10.1165/rcmb.2008-0423OC 19617401 PMC2891499

[32] Singh B, Bogatcheva G, Washington MK, Coffey RJ. 2013. Transformation of polarized epithelial cells by apical mistrafficking of epiregulin. Proc Natl Acad Sci USA 110:8960–8965. doi:10.1073/pnas.1305508110 23671122 PMC3670353

[33] Riese DJ II, Cullum RL. 2014. Epiregulin: roles in normal physiology and cancer. Seminars in Cell & Developmental Biology 28:49–56. doi:10.1016/j.semcdb.2014.03.005 24631357 PMC4037385

[34] van Tol S, Hage A, Giraldo MI, Bharaj P, Rajsbaum R. 2017. The TRIMendous role of TRIMs in virus-host interactions. Vaccines (Basel) 5:23. doi:10.3390/vaccines5030023 28829373 PMC5620554

[35] Job ER, Bottazzi B, Short KR, Deng YM, Mantovani A, Brooks AG, Reading PC. 2014. A single amino acid substitution in the hemagglutinin of H3N2 subtype influenza A viruses is associated with resistance to the long pentraxin PTX3 and enhanced virulence in mice. J Immunol 192:271–281. doi:10.4049/jimmunol.1301814 24307735

[36] Reading PC, Bozza S, Gilbertson B, Tate M, Moretti S, Job ER, Crouch EC, Brooks AG, Brown LE, Bottazzi B, Romani L, Mantovani A. 2008. Antiviral activity of the long chain pentraxin PTX3 against influenza viruses. J Immunol 180:3391–3398. doi:10.4049/jimmunol.180.5.3391 18292565

[37] König R, Stertz S, Zhou Y, Inoue A, Hoffmann H-H, Bhattacharyya S, Alamares JG, Tscherne DM, Ortigoza MB, Liang Y, et al.. 2010. Human host factors required for influenza virus replication. Nature 463:813–817. doi:10.1038/nature08699 20027183 PMC2862546

[38] Coleman MD, Ha SD, Haeryfar SMM, Barr SD, Kim SO. 2018. Cathepsin B plays a key role in optimal production of the influenza A virus. J Virol Antivir Res 7:1–20. doi:10.4172/2324-8955.1000178 29349092 PMC5770218

[39] Teng O, Chen ST, Hsu TL, Sia SF, Cole S, Valkenburg SA, Hsu TY, Zheng JT, Tu W, Bruzzone R, Peiris JSM, Hsieh SL, Yen HL. 2017. CLEC5A-mediated enhancement of the inflammatory response in myeloid cells contributes to influenza virus pathogenicity in vivo. J Virol 91:e01813-16. doi:10.1128/JVI.01813-16 27795434 PMC5165214

[40] Falcon A, Cuevas MT, Rodriguez-Frandsen A, Reyes N, Pozo F, Moreno S, Ledesma J, Martínez-Alarcón J, Nieto A, Casas I. 2015. CCR5 deficiency predisposes to fatal outcome in influenza virus infection. J Gen Virol 96:2074–2078. doi:10.1099/vir.0.000165 25918237

[41] Rudd JM, Pulavendran S, Ashar HK, Ritchey JW, Snider TA, Malayer JR, Marie M, Chow VTK, Narasaraju T. 2019. Neutrophils induce a novel chemokine receptors repertoire during influenza pneumonia. Front Cell Infect Microbiol 9:108. doi:10.3389/fcimb.2019.00108 31041196 PMC6476945

[42] Nagesh PT, Husain M. 2016. Influenza A virus dysregulates host histone deacetylase 1 that inhibits viral infection in lung epithelial cells. J Virol 90:4614–4625. doi:10.1128/JVI.00126-16 26912629 PMC4836332

[43] Vroman H, Das T, Bergen IM, van Hulst JAC, Ahmadi F, van Loo G, Lubberts E, Hendriks RW, Kool M. 2018. House dust mite-driven neutrophilic airway inflammation in mice with TNFAIP3-deficient myeloid cells is IL-17-independent. Clin Exp Allergy 48:1705–1714. doi:10.1111/cea.13262 30171721

[44] Geoffroy MC, Chelbi-Alix MK. 2011. Role of promyelocytic leukemia protein in host antiviral defense. J Interferon Cytokine Res 31:145–158. doi:10.1089/jir.2010.0111 21198351

[45] Shapira SD, Gat-Viks I, Shum BOV, Dricot A, de Grace MM, Wu L, Gupta PB, Hao T, Silver SJ, Root DE, Hill DE, Regev A, Hacohen N. 2009. A physical and regulatory map of host-influenza interactions reveals pathways in H1N1 infection. Cell 139:1255–1267. doi:10.1016/j.cell.2009.12.018 20064372 PMC2892837

[46] Wilk AJ, Lee MJ, Wei B, Parks B, Pi R, Martínez-Colón GJ, Ranganath T, Zhao NQ, Taylor S, Becker W, Jimenez-Morales D, Blomkalns AL, O’Hara R, Ashley EA, Nadeau KC, Yang S, Holmes S, Rabinovitch M, Rogers AJ, Greenleaf WJ, Blish CA, Stanford COVID-19 Biobank. 2021. Multi-omic profiling reveals widespread dysregulation of innate immunity and hematopoiesis in COVID-19. J Exp Med 218:e20210582. doi:10.1084/jem.20210582 34128959 PMC8210586

[47] Akram KM, Moyo NA, Leeming GH, Bingle L, Jasim S, Hussain S, Schorlemmer A, Kipar A, Digard P, Tripp RA, Shohet RV, Bingle CD, Stewart JP. 2018. An innate defense peptide BPIFA1/SPLUNC1 restricts influenza A virus infection. Mucosal Immunol 11:71–81. doi:10.1038/mi.2017.45 28513596

[48] Lu X, Masic A, Liu Q, Zhou Y. 2011. Regulation of influenza A virus induced CXCL-10 gene expression requires PI3K/Akt pathway and IRF3 transcription factor. Mol Immunol 48:1417–1423. doi:10.1016/j.molimm.2011.03.017 21497908

[49] Wang W, Yang P, Zhong Y, Zhao Z, Xing L, Zhao Y, Zou Z, Zhang Y, Li C, Li T, Wang C, Wang Z, Yu X, Cao B, Gao X, Penninger JM, Wang X, Jiang C. 2013. Monoclonal antibody against CXCL-10/IP-10 ameliorates influenza A (H1N1) virus induced acute lung injury. Cell Res 23:577–580. doi:10.1038/cr.2013.25 23419516 PMC3616436

[50] Karasik A, Jones GD, DePass AV, Guydosh NR. 2021. Activation of the antiviral factor RNase L triggers translation of non-coding mRNA sequences. Nucleic Acids Res 49:6007–6026. doi:10.1093/nar/gkab036 33556964 PMC8216459

[51] Sarkar L, Liu G, Gack MU. 2023. ISG15: its roles in SARS-CoV-2 and other viral infections. Trends Microbiol 31:1262–1275. doi:10.1016/j.tim.2023.07.006 37573184 PMC10840963

[52] Tripathi S, Batra J, Cao W, Sharma K, Patel JR, Ranjan P, Kumar A, Katz JM, Cox NJ, Lal RB, Sambhara S, Lal SK. 2013. Influenza A virus nucleoprotein induces apoptosis in human airway epithelial cells: implications of a novel interaction between nucleoprotein and host protein Clusterin. Cell Death Dis 4:e562–e562. doi:10.1038/cddis.2013.89 23538443 PMC3615740

[53] Tang Z, Mao Y, Ruan P, Li J, Qiu X, Meng Y, Wang M, Wu G, Wang L, Tan Y. 2024. Drugs targeting CMPK2 inhibit pyroptosis to alleviate severe pneumonia caused by multiple respiratory viruses. J Med Virol 96:e29643. doi:10.1002/jmv.29643 38695269

[54] Chiou SJ, Wang CC, Tseng YS, Lee YJ, Chen SC, Chou CH, Chuang LY, Hong YR, Lu CY, Chiu CC, Chignard M. 2016. A novel role for β2-microglobulin: a precursor of antibacterial chemokine in respiratory epithelial cells. Sci Rep 6:31035. doi:10.1038/srep31035 27503241 PMC4977529

[55] Ardeniz Ö, Unger S, Onay H, Ammann S, Keck C, Cianga C, Gerçeker B, Martin B, Fuchs I, Salzer U, İkincioğulları A, Güloğlu D, Dereli T, Thimme R, Ehl S, Schwarz K, Schmitt-Graeff A, Cianga P, Fisch P, Warnatz K. 2015. beta2-Microglobulin deficiency causes a complex immunodeficiency of the innate and adaptive immune system. J Allergy Clin Immunol 136:392–401. doi:10.1016/j.jaci.2014.12.1937 25702838

[56] He J, Sun E, Bujny MV, Kim D, Davidson MW, Zhuang X. 2013. Dual function of CD81 in influenza virus uncoating and budding. PLoS Pathog 9:e1003701. doi:10.1371/journal.ppat.1003701 24130495 PMC3795033

[57] Stading R, Couroucli X, Lingappan K, Moorthy B. 2021. The role of cytochrome P450 (CYP) enzymes in hyperoxic lung injury. Expert Opin Drug Metab Toxicol 17:171–178. doi:10.1080/17425255.2021.1853705 33215946 PMC7864879

[58] Lingappan K, Maity S, Jiang W, Wang L, Couroucli X, Veith A, Zhou G, Coarfa C, Moorthy B. 2017. Role of cytochrome P450 (CYP)1A in hyperoxic lung injury: analysis of the transcriptome and proteome. Sci Rep 7:642. doi:10.1038/s41598-017-00516-x 28377578 PMC5428698

[59] Szostecki C, Guldner HH, Netter HJ, Will H. 1990. J Immunol 145:4338–4347. doi:10.4049/jimmunol.145.12.4338 2258622

[60] Ma Y, Li J, Dong H, Yang Z, Zhou L, Xu P. 2022. PML body component Sp100A restricts wild-type herpes simplex virus 1 infection. J Virol 96:e0027922. doi:10.1128/jvi.00279-22 35353002 PMC9044927

[61] Zaraket H, Baranovich T, Kaplan BS, Carter R, Song MS, Paulson JC, Rehg JE, Bahl J, Crumpton JC, Seiler J, Edmonson M, Wu G, Karlsson E, Fabrizio T, Zhu H, Guan Y, Husain M, Schultz-Cherry S, Krauss S, McBride R, Webster RG, Govorkova EA, Zhang J, Russell CJ, Webby RJ. 2015. Mammalian adaptation of influenza A(H7N9) virus is limited by a narrow genetic bottleneck. Nat Commun 6:6553. doi:10.1038/ncomms7553 25850788 PMC4403340

[62] Alnaji FG, Holmes JR, Rendon G, Vera JC, Fields CJ, Martin BE, Brooke CB. 2019. Sequencing framework for the sensitive detection and precise mapping of defective interfering particle-associated deletions across influenza A and B viruses. J Virol 93:e00354-19. doi:10.1128/JVI.00354-19 30867305 PMC6532088

[63] Saira K, Lin X, DePasse JV, Halpin R, Twaddle A, Stockwell T, Angus B, Cozzi-Lepri A, Delfino M, Dugan V, Dwyer DE, Freiberg M, Horban A, Losso M, Lynfield R, Wentworth DN, Holmes EC, Davey R, Wentworth DE, Ghedin E, Group IFS, Group IFS. 2013. Sequence analysis of in vivo defective interfering-like RNA of influenza A H1N1 pandemic virus. J Virol 87:8064–8074. doi:10.1128/JVI.00240-13 23678180 PMC3700204

[64] Seth RB, Sun L, Ea CK, Chen ZJ. 2005. Identification and characterization of MAVS, a mitochondrial antiviral signaling protein that activates NF-kappaB and IRF 3. Cell 122:669–682. doi:10.1016/j.cell.2005.08.012 16125763

[65] Yoshizumi T, Imamura H, Taku T, Kuroki T, Kawaguchi A, Ishikawa K, Nakada K, Koshiba T. 2017. RLR-mediated antiviral innate immunity requires oxidative phosphorylation activity. Sci Rep 7:5379. doi:10.1038/s41598-017-05808-w 28710430 PMC5511143

[66] Bissel SJ, Carter CE, Wang G, Johnson SK, Lashua LP, Kelvin AA, Wiley CA, Ghedin E, Ross TM. 2019. Age-related pathology associated with H1N1 A/California/07/2009 influenza virus infection. Am J Pathol 189:2389–2399. doi:10.1016/j.ajpath.2019.08.017 31585069 PMC6893900

