# Supplementary material for: Inflammatory, transcriptomic, and cell fate responses underlying the mammalian transmission of avian influenza viruses
Source: J Virol. 2025 Aug 8;99(9):e00647-25. doi: 10.1128/jvi.00647-25 (PMC12456136; doi:10.1128/jvi.00647-25)
Supplement: Supplemental material — Figures S1 to S3; Tables S1 and S2. [file jvi.00647-25-s0001.pdf]

Supplementary material

Supplementary Figure 1.

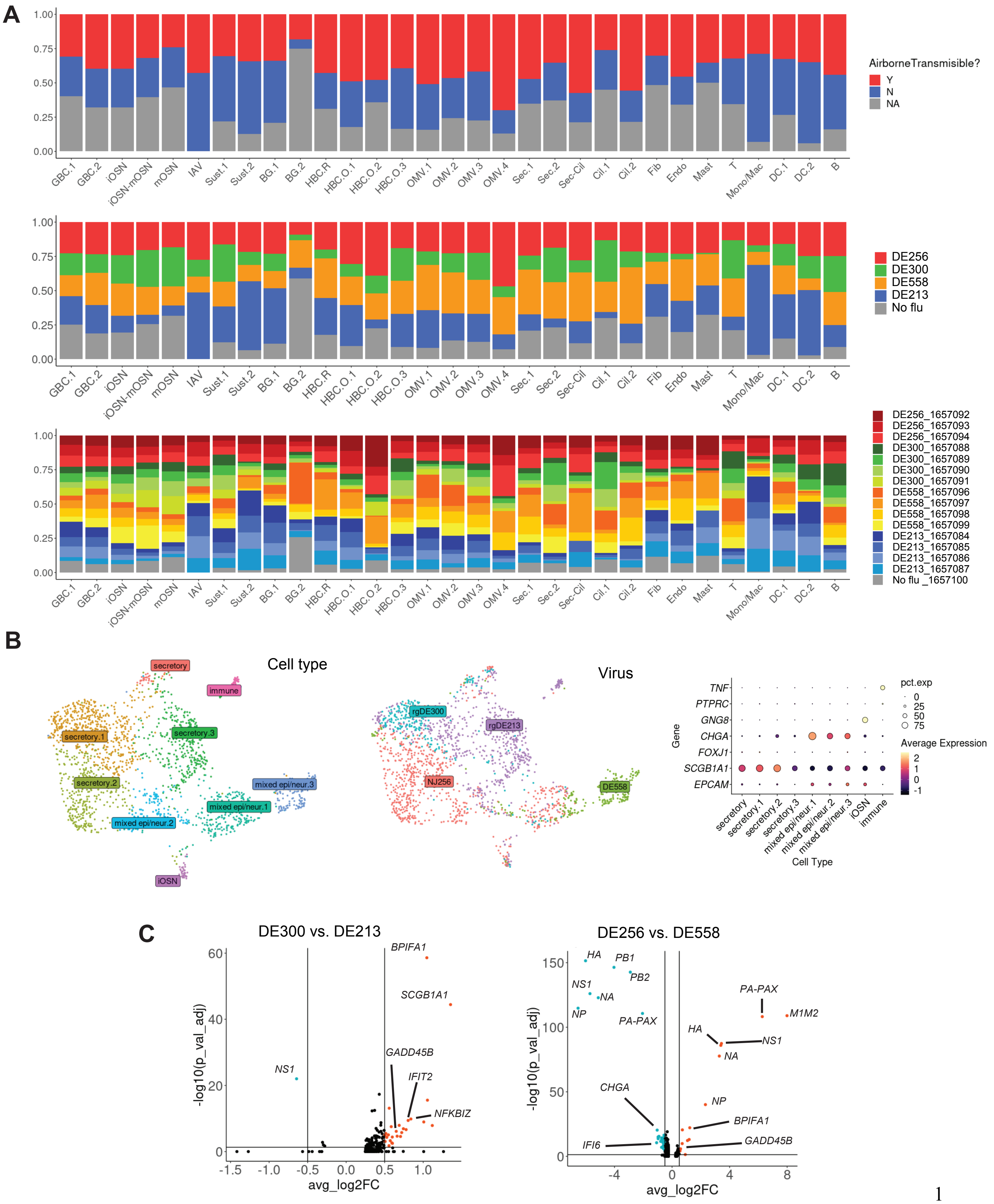

Supplementary Figure 2.

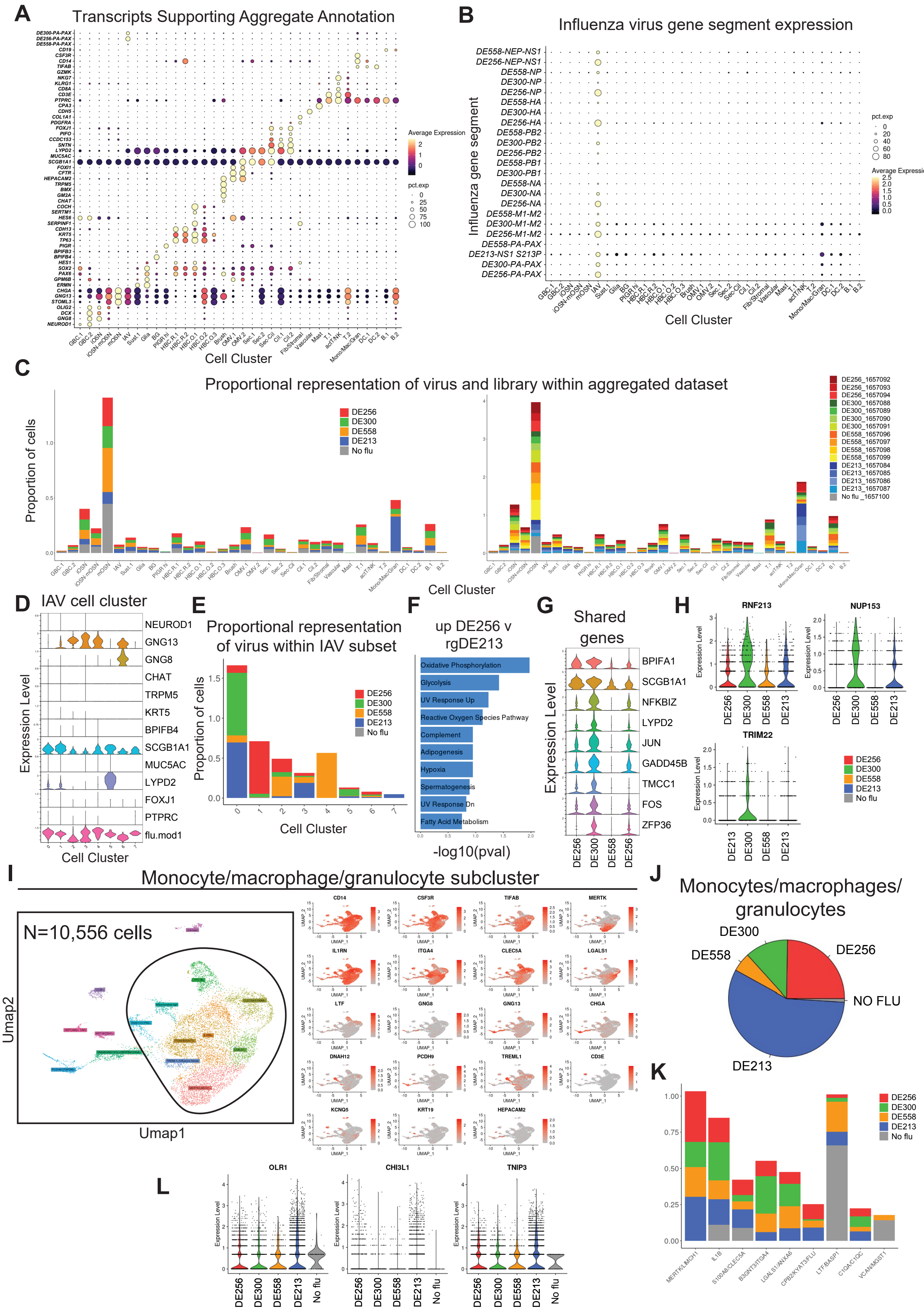

Supplementary Figure 3.

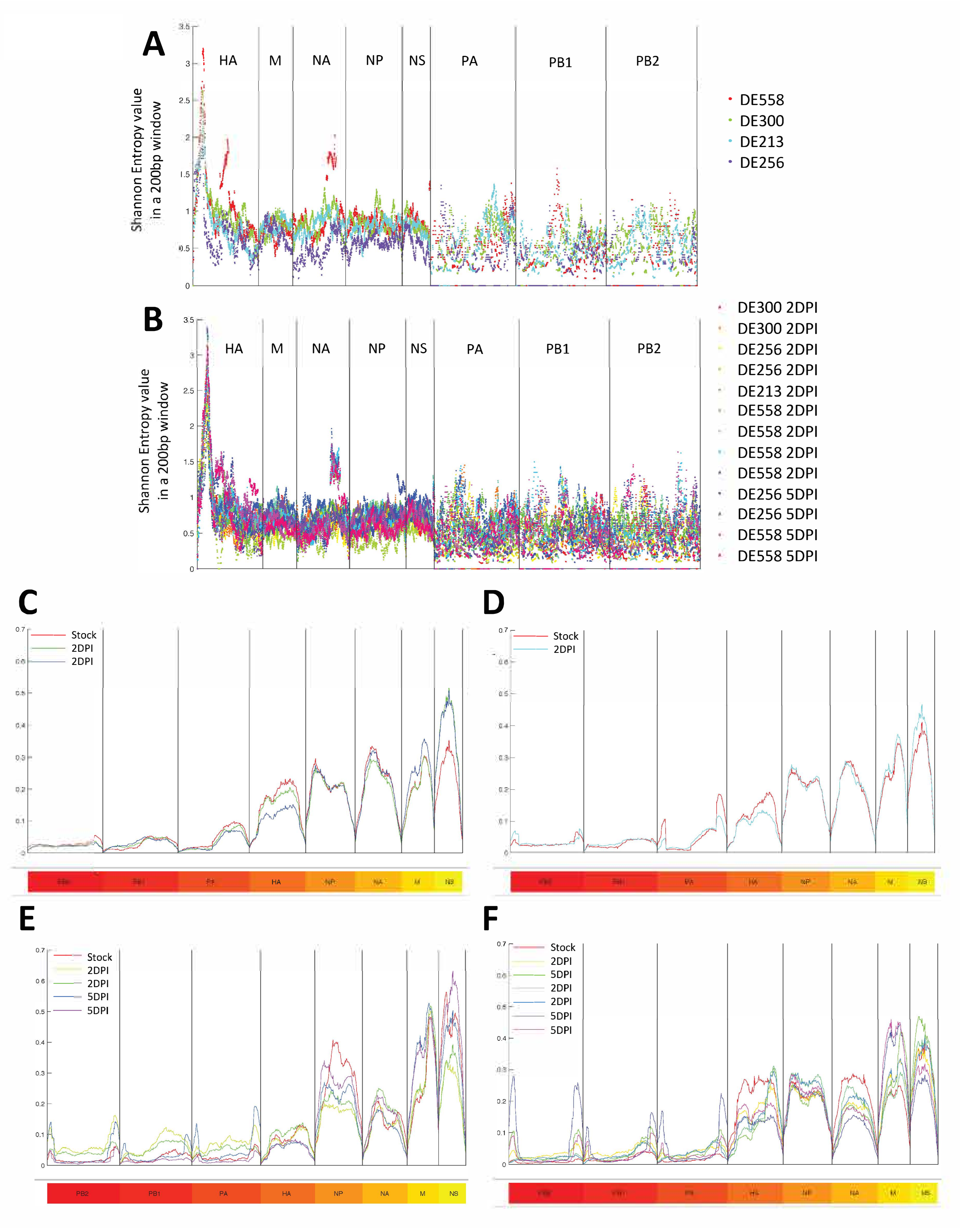

Supplementary Figure Legends.

Supplementary Figure 1. Supporting data for aggregate and subclustered single cell datasets. (A) stacked bar plots representing proportions of cells grouped by transmissibility (top), virus (middle), and animal (bottom) within individual cell populations present in aggregate data (as in Figures 3A and 1B). (B) UMAPs of subclustered IAV infected cells ("IAV" in Figures 3A and 1B) labelled by cell type (top left) and virus (bottom left) and dot plot demonstrating expression of select transcripts by individual cell populations within the subclustered IAV infected cells (right). The size of the dot corresponds to the percent of cells within a population expressing a transcript and the color of the dot corresponds to the average expression of the transcript within the cell population. (C) Volcano plots showing differentially expressed transcripts when comparing cells from rgDE300 and rgDE213 cells (left) and DE256 and DE558 (right) within the subclustered IAV infected cell population. Horizontal lines indicate an adjusted p-value of 0.05 and vertical lines indicate an avg_log2 Fold Change (FC) of 0.5. Positive fold changes indicate differential upregulation by rgDE300 and DE256 (left and right volcano plots, respectively).

Supplementary Figure 2. Single cell RNA sequencing data. (A) Dot plot of cell type and cell state specific transcripts supporting aggregate clustering annotation (as in Figure 3A). The size of the dot shows the percentage of cells within a cell cluster and the color represents the average expression level across all cells within a cluster. (B) Dot plot demonstrating expression of each individual influenza gene segment from the viruses used to inoculate ferrets in this study within each cell cluster in aggregate. The size of the dot shows the percentage of cells within a cell cluster and the color represents the average expression level across all cells within a cluster. (C) Stacked bar plots demonstrating the proportion of cells from ferrets infected with an individual virus present within an individual cell cluster (left) and proportion of cells from individual single cell gene expression library present within an individual cell cluster (right). (D) Stacked violin plot demonstrating expression of individual transcripts in cell clusters present in the subsetted and reclustered IAV cell cluster. "flu.mod1" represents the usage of a gene list composed of individual influenza gene segments included in the custom reference to which the sequencing data were aligned. (E) Stacked bar plots demonstrating the proportion of cells from ferrets infected with an individual virus present within an individual IAV cell cluster. (F) Bar plot demonstrating top 10 enriched MSigDB_Hallmark_2020 terms comparing NJ256 cells to DE213 cells within the subsetted and reclustered IAV

population. (G) Stacked violin plot demonstrating expression levels of individual transcripts (y-axis) within the subsetted and reclustered IAV population, grouped by infecting virus name (x-axis). (H) Violin plots of three transcripts differentially upregulated between DE300 and DE213 in differential gene expression analysis within the subsetted and reclustered IAV population, grouped by infecting virus name (x-axis). (I) Uniform manifold approximation and projection (UMAP) demonstrating subsetted and reclustered monocyte/macrophage/granulocyte cells (subsetted from aggregate data, Figure 3A, left) and array of feature plots demonstrating expression level of individual transcripts amongst subsetted and reclustered monocyte/macrophage/granulocyte cells (right). For feature plots, cells are colored according to expression of each individual transcript. (J) Pie chart demonstrating the proportion of total mono/mac/gran (n=9205 cells as in Figure 3F) cells from ferrets infected with each individual virus. (K) Stacked bar plot demonstrating the proportion of cells from ferrets infected with an individual virus present within an individual subsetted and reclustered mono/mac/gran (n = 9205 cells as in Figure 3F) cell cluster. (L) Violin plots of three transcripts differentially regulated between DE300 and DE213 in differential gene expression analysis within mono/mac/gran cells (n = 9205 cells as in Figure 3F) grouped by infecting virus name (x-axis).

Supplementary figure 3. Sequence coverage and hot spots of Shannon Entropy complexity among viral populations in ferret nasal washes. Shannon Entropy complexity within a 200bp sliding window among eight viral genes are shown of the viral stocks used to inoculate ferrets (A) and viral populations in ferret nasal washes collected at two- and five-days post infection (B). Coverage maps (C-F) show the average number of reads mapped to a 50bp sliding window in each viral gene normalized by the total number of reads mapped to each gene. Coverage maps revealed the likely presence of defective interfering particles in the polymerase genes of DE213, DE256 and DE558 (D, E and F, respectively) but not DE300 (C).

Supplementary tables

Supplementary table 1. Amino acid differences associated with different mammalian transmissibility phenotypes in the viruses used in this study.

Virus name[a]	PB2					PB1		PB1-F2									PA-X	NS1			NEP	
Amino acid	67	152	199	508	649	298	642	667	8	15	23	26	27	31	58	69	75	21	7	213	227	7	70
DE256	V	S	T	Q	I	I	S	V	Q	R	S	R	I	G	S	R	L	V	L	S	G	L	G
DE300	V	S	T	Q	I	I	S	V	Q	R	S	R	I	G	S	R	L	V	L	S	G	L	G
DE213	V	S	T	Q	I	I	S	V	Q	R	S	R	I	G	S	R	L	V	L	P	G	L	G
DE558	I	A	A	R	V	L	N	I	P	H	N	Q	T	E	L	Q	R	A	S	P	E	S	S

[a]DE256 - A/ruddy turnstone/New Jersey/AI09_256/2009 (H1N1)
DE300 – A/ruddy turnstone/Delaware/300/2009 (H1N1) (produced by reverse genetics)
DE213 – A/ruddy turnstone/Delaware/300/2009 (S213P NS1) (H1N1) (produced by reverse genetics)
DE558 – A/shorebird/Delaware Bay/558/2006 (H1N1)

Supplementary table 2. Findings of *in situ* hybridization in the ferret nasal epithelium.

Target	DE256	DE558
LOC101067500	Diffuse labeling of respiratory epithelium, extensive labeling of sustentacular cells in olfactory epithelium.	Diffuse labeling of respiratory epithelium, extensive labeling of sustentacular cells in olfactory epithelium and submucosal glands
OAS2	No labeling	No labeling
B2M	Inconsistent labeling	Multifocal labeling of respiratory and olfactory epithelia.
CMPK2	Extensive labeling of the respiratory and olfactory epithelia plus other tissues including the periosteum, endothelium, glands and iris.	Multifocal labeling of respiratory and olfactory epithelia
CD81	Inconsistent labeling	Inconsistent labeling
CLU	Strong staining of submucosal glands, sustentacular cells in olfactory epithelium, goblet cells in respiratory epithelium.	Strong staining of submucosal glands, sustentacular cells in olfactory epithelium, goblet cells in respiratory epithelium.
SP100	Diffuse labeling of respiratory epithelium and extensive labeling of olfactory epithelium.	Multifocal labeling of respiratory and olfactory epithelia.
CXCL10	Strong labeling of respiratory epithelium and associated submucosa.	Patchy labeling of turbinates, respiratory epithelium plus some labeling of sustentacular cells in olfactory epithelium.
ISG15	Extensive intense labeling of olfactory and respiratory epithelium and associated submucosa.	Extensive strong labeling of olfactory and respiratory epithelium and associated submucosa.
BPIFA1	Diffuse, intense labeling of respiratory epithelium and scattered labeling of cells in the olfactory neuroepithelium.	Diffuse intense labeling of respiratory epithelium and scattered labeling of cells in the olfactory neuroepithelium.
